# Enhancing the Extraction of Polysaccharides and Antioxidants from Macroalgae Using Sequential Hydrothermal-Assisted Extraction Followed by Ultrasound and Thermal Technologies

**DOI:** 10.3390/md17080457

**Published:** 2019-08-05

**Authors:** Marco Garcia-Vaquero, John V. O’Doherty, Brijesh K. Tiwari, Torres Sweeney, Gaurav Rajauria

**Affiliations:** 1 School of Veterinary Medicine, Veterinary Science Centre, University College Dublin, Belfield, Dublin 4, Ireland; 2 School of Agriculture and Food Science, University College Dublin, Lyons Research Farm, Celbridge, Co. Kildare W23 ENY2, Ireland; 3 TEAGASC, Food Research Centre, Ashtown, Dublin D15 KN3K, Ireland

**Keywords:** fucoidan, glucan, laminarin, carbohydrate, innovative technology, biorefinery, functional food, optimisation, response surface methodology

## Abstract

Fucose sulphated polysaccharides (FSPs) and glucans have recently attracted the attention of the scientific community due to their wide range of biological activities. Both polysaccharides should ideally be selectively extracted using innovative technologies with high extraction efficiency. This study aims to: (1) Optimise the extraction variables used in hydrothermal-assisted extraction (HAE) to obtain high yields of FSPs, total glucans, and antioxidants from *Laminaria hyperborea*; (2) to apply these optimised protocols to other brown macroalgae; and (3) to explore the application of ultrasound and thermal technologies to increase the recovery of polysaccharides from the residual biomass. Box-Behnken design (three-factor, four-levels) was employed to optimise the HAE variables, and principal component analysis was used to evaluate the recovery of polysaccharides from the residual biomass. The optimal HAE conditions were 120 °C, 80.9 min, and 12.02 mL/g macroalgae from *L. hyperborea*. The best sequential application of ultrasound and thermal treatment achieved an additional 2971.7 ± 61.9 mg fucose/100 g dried macroalgal residue (dmr) from *Ascophyllum nodosum* and 908.0 ± 51.4 mg total glucans/100 g dmr from *L. hyperborea* macroalgal residues.

## 1. Introduction

Macroalgae comprises a large and diverse group of aquatic plants able to adapt to extreme environmental conditions by producing unique secondary metabolites, such as polysaccharides [1,2]. Macroalgae have been traditionally used as a source of carbohydrates, mainly hydrocolloids such as carrageenan, agars, and alginates, with physico-chemical properties (i.e., gelling, thickening, and stabilising properties) that are useful for the development of food, cosmetic, and pharmaceutical formulations [3,4,5]. Moreover, other polysaccharides from brown macroalgae such as fucose sulphated polysaccharides (FSPs) and glucans (i.e., β-glucans or laminarin) have been explored for the development of new therapeutic drugs, functional foods, or nutraceuticals [6,7,8].

FSPs are a heterogeneous group of carbohydrates produced mainly by brown macroalgae and marine invertebrates [7]. In brown macroalgae, FSPs can be found binding strongly to other carbohydrates (i.e., alginate, cellulose, and hemicellulose) of the cell wall, in order to maintain cellular integrity [7]. The main chemical structure of FSPs consist of α-(1-3) or alternating α-(1-3) with α-(1-4) linear or branched L-fucopyranose backbones, with other minor monosaccharide constituents including galactose, mannose, xylose, and glucose, depending on the macroalgal species [9].

Glucans are the main storage or energy reserve carbohydrates produced by brown macroalgae [10]. These polysaccharides, mainly β-glucans or laminarin, are located in vacuoles inside the cells and can constitute up to 35% of the dried weight of the biomass in certain macroalgae species [6,11]. The chemical structure of these compounds has been described as a backbone of β-1,3-linked d-glucose units with variable degrees of branching (positions 6 and 2), although the molecules may contain variable amounts of other monosaccharides substituents, mainly mannitol [10,12].

FSPs and glucans have recently attracted the attention of the scientific community due to their wide range of biological activities [6,13] without side effects of these macroalgal carbohydrates, compared to other drugs when used in biomedical applications [7]. Both polysaccharides have shown beneficial effects when used as anti-inflammatory, anticoagulant, antioxidant, antiviral, anti-tumour, anti-obesity, and antimicrobial agents in vitro and in vivo [6,11,13,14,15]. The biological activities of FSPs and glucans are related to the chemical structure of the molecules, which vary significantly depending on the macroalgae (i.e., species, parts of the algae, and biological stage of the biomass) and on other environmental factors affecting the biomass, such as season and location of collection [12,16]. Moreover, the extraction procedures used to obtain macroalgal polysaccharides could also greatly influence the chemical structure and activity of the molecules [6,7,8,15]. In order to fully utilise the macroalgal biomass, both polysaccharides should ideally be selectively extracted, achieving high yields of compounds by using innovative technologies aiming to improve the efficiency of the extraction while reducing the generation of waste and the use of chemicals [17]. Thus, there is a need to design novel extraction protocols to optimise the extraction conditions of polysaccharides from macroalgae focusing on the yields, but also on the biological activities of the compounds [6,15,18]. Rajauria et al. [19] used autoclave-based hydrothermal-assisted extraction (HAE) to obtain carbohydrates and other phytochemicals from macroalgae, emphasising the promising applications of this HAE technology beyond its current main use in food processing. However, the authors did not optimise the main extraction variables.

Moreover, after performing an initial extraction, most researchers collect the polysaccharide-rich extracts by filtering or centrifuging the biomass, and repeat the extraction procedures on the macroalgal residues, using one or several extraction cycles or multiple technologies to increase the yields of FSPs and glucans. Shan et al. [20] generated extracts rich in FSPs with antidiabetic properties from five brown macroalgae using three extraction cycles (80 °C, 3 h), and collected the macroalgal residues by centrifugation after every extraction. Understanding the impact of multiple process parameters on the efficiency of the extraction and yields of carbohydrates and other high-value compounds is one of the key critical points that will influence the design and utilisation of macroalgal biomass, following a biorefinery concept at industrial scale [21,22].

This study aims to use statistical optimisation tools such as the multivariate statistic technique, response surface methodology (RSM), to: (1) Optimise the effect of HAE variables (temperature, time, and volume of solvent) to obtain high yields of FSPs (estimated as fucose) and total glucans with potent antioxidant activities (ferric reducing antioxidant power (FRAP) and 1,1-diphenyl-2-picryl-hydrazyl (DPPH)) from *L. hyperborea*; and (2) to explore these optimum HAE conditions in other brown macroalgae (*Laminaria digitata* and *A. nodosum*). Following HAE, the residues were re-extracted, aiming to elucidate the impact of the combination of ultrasound and thermal technologies sequentially on the yields of fucose and total glucans that could be applied at industrial scale.

## 2. Results and Discussion

### 2.1. Proximate Composition of Macroalgae

The proximate composition of the dried macroalgal biomass used in later extraction procedures is summarised in Table 1.

### 2.2. Modelling the Extraction Variables of Hydrothermal-Assisted Extraction (HAE)

**Figure 1:**
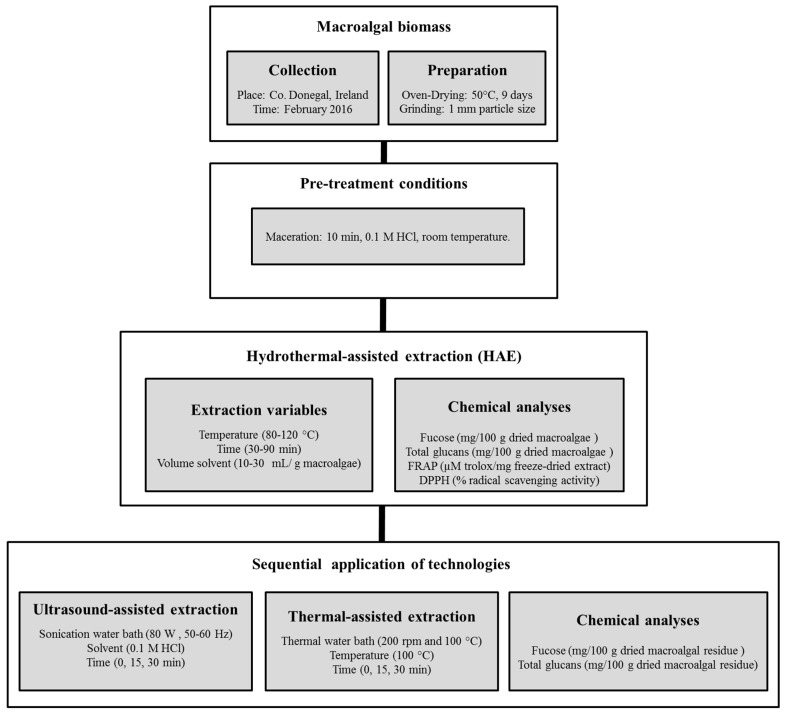
Scheme detailing the pre-treatments of the fresh macroalgae, the hydrothermal-assisted extraction conditions used to generate macroalgal extracts, and the sequential application of ultrasound and thermal technologies to increase the recovery of compounds from the residual biomass.

The scheme of work followed to generate extracts using HAE from *L. hyperborea* and the sequential extraction of the residual biomass using ultrasound and thermal extraction technologies is represented in Figure 1, with further details explained in point 3.3 of materials and methods.

The matrix design and experimental responses for fucose, total glucans, FRAP, and DPPH obtained from *L. hyperborea* are summarised in Table 2. The experimental responses were very variable between the different experimental runs: Fucose (ranging from 1161.06 to 3216.39 mg/100 g dried macroalgae (dm)), total glucans (1571.88 to 3325.41 mg/100 g dm), FRAP (17.34 to 59.64 µM trolox/mg freeze-dried extract (fde)) and DPPH activities (11.69 to 45.34%). The highest yields of fucose (3216.39 mg/100 g dm) were obtained at 120 °C, 60 min and 30 mL of solvent per gram of macroalgae, while the concentration of total glucans was maximum (3325.41 mg/100 g dm) when applying lower temperatures (100 °C) for 60 min and using less solvent (20 mL per gram of macroalgae). Both antioxidant activities, FRAP (59.64 µM trolox/mg fde) and DPPH (45.34%), were maximised by using 120 °C, 60 min, and 10 mL of solvent per gram of macroalgae.

A second order polynomial model fitted well to the experimental data (see Table 3) with low standard error and regression co-efficient (R^2^) values equal to or higher than 0.80 for all the parameters of interest. ANOVA for the response surfaces showed that the linear models were significant for fucose (*p* < 0.01) and FRAP (*p* < 0.05), and tended to be significant (*p* < 0.1) for DPPH, while the quadratic model was significant for total glucans (*p* < 0.01) and tended towards statistical significance (*p* < 0.1) for both FRAP and DPPH. Moreover, the linear and quadratic models caused significant effects on the response surfaces, and the total model was significant (*p* < 0.05) for all the parameters analysed, with a tendency towards significance (*p* < 0.1) for DPPH. The cross-products or interactions among the extraction parameters in this study were not significant.

The significance of the experimental variables affecting the extraction of polysaccharides and antioxidant activity of extracts generated from *L. hyperborea* using HAE can be assessed by the model coefficients obtained from the response surface regression and ANOVA analysis compiled in Table 4. The magnitude of each coefficient is related to the weight of its effect and the signs indicate an increase (+) and decrease (−) in the experimental responses. The constant coefficient (β_0_) had an influence (*p* < 0.05) on the levels of total glucans and DPPH. The temperature (β_1_) influenced (*p* < 0.05) the extraction of total glucans and DPPH and tended to influence (*p* < 0.1) the FRAP antioxidant activities of extracts from *L. hyperborea*, while the volume of solvent (β_3_) influenced (*p* < 0.01) the extraction of total glucans. The quadratic effect of temperature (β_11_) influenced (*p* < 0.05) the extraction of total glucans, FRAP, and DPPH; while the solvent (β_33_) influenced (*p* < 0.01) the extraction of total glucans from *L. hyperborea*. No interactions or cross-products were appreciated for any experimental response, with tendencies towards statistical significance (*p* < 0.1) in the case of total glucans and FRAP (temperature and solvent, β_13_).

The Equations (1)–(4) describe the influence of the experimental variables temperature (X_1_), time (X_2_), and volume of solvent (X_3_) on the yields of fucose, total glucans, and antioxidant activities (FRAP and DPPH) from *L. hyperborea* and can be accessed in the supplementary material of this manuscript.

Fucose (mg/100 g dm) = −54 − 25.4 X1 + 47.7 X2 + 48 X3 + 0.199 X1 X1 − 0.354 X2 X2 − 1.43 X3 X3 − 0.009 X1 X2 + 0.450 X1 X3 − 0.095 X2 X3(1)

Total glucans (mg/100 g dm) = −12682 + 233.4 X1 − 40.7 X2 + 471 X3 − 1.033 X1 X1 + 0.086 X2 X2 − 6.95 X3 X3 + 0.301 X1 X2 − 1.733 X1 X3 − 0.076 X2 X3(2)

FRAP (µM trolox/mg fde) = 122.6 − 2.75 X1 + 0.186 X2 + 1.81 X3 + 0.01739 X1 X1 − 0.00531 X2 X2 + 0.022 X3 X3 + 0.00498 X1 X2 − 0.0331 X1 X3 + 0.00111 X2 X3(3)

DPPH (% radical scavenging activity) = 261.5 − 4.2 X1 − 0.732 X2 − 0.44 X3 + 0.02242 X1 X1 − 0.00225 X2 X2 + 0.0591 X3 X3 + 0.00414 X1 X2 − 0.0304 X1 X3 + 0.0072 X2 X3(4)

**Figure 2:**
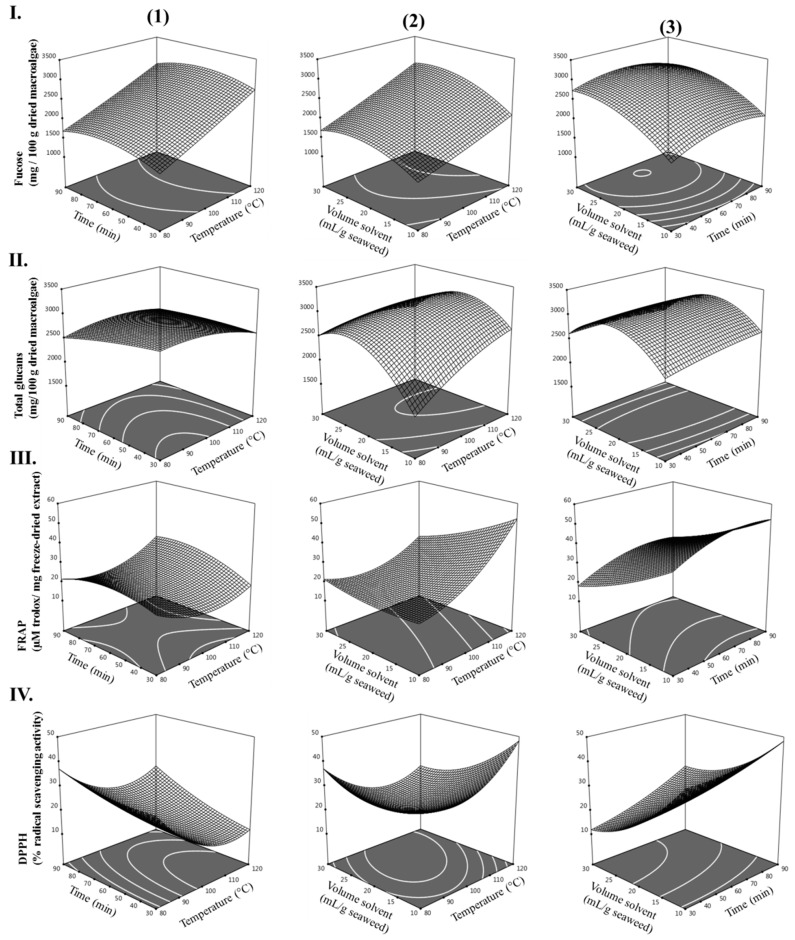
Contour plots (2D) and response surface plots (3D) of (**I**) fucose (mg/100 g dried macroalgae); (**II**) total glucans (mg/100 g dried macroalgae); (**III**) FRAP (µM trolox/mg freeze-dried extract); and (**IV**) DPPH (% radical scavenging activity) extracted from *L. hyperborea* as a function of (**1**) time to temperature (volume solvent = 30 mL/g macroalgae), (**2**) volume of solvent to temperature (time = 120 min), and (**3**) volume of solvent to time (temperature = 120 °C).

Mixed 2D–3D plots, illustrating the influence of the experimental variables (temperature, time, and volume of solvent) on the experimental responses (fucose, total glucans, FRAP, and DPPH) of extracts from *L. hyperborea* were generated from the model equations (see Figure 2) and used to predict the optimum HAE conditions for *L. hyperborea*. Each graphic represents the effect of two extraction variables on the levels of fucose, total glucans, FRAP, and DPPH, while keeping the non-represented variable at its maximum.

### 2.3. Optimum HAE Conditions

Optimum HAE conditions were designed using the surface regression model equations based on the experimental data for optimisation (Table 3). The optimum conditions aiming to maximise the yields of fucose (condition 1), total glucans (condition 2), antioxidant activities (FRAP and DPPH) (condition 3), and all the previous experimental responses (fucose, total glucans, FRAP, and DPPH) combined (condition 4) are summarised in Table 5. The temperature, time, and volume of solvent needed for each optimised condition, the predicted values of the theoretical model, and the experimental responses obtained when performing the extraction using *L. hyperborea* are compiled in Table 5. The experimental data confirmed the predicted values of the model for fucose, total glucans, FRAP, and DPPH for all the optimum conditions, with the exception of the experimental fucose in condition 4 that slightly exceeded the predicted values of the model.

The extraction conditions used to maximise the yields of fucose from *L. hyperborea* were 120 °C, 62.1 min, and 30 mL of solvent per gram of macroalgae (see condition 1 in Table 5). Wang et al. [23] used HAE at 120 °C, 3 h, and 35 mL of solvent per gram of macroalgae to extract fucose from *Laminaria japonica;* however, this extraction protocol was not optimised. Saravana et al. [24] optimised HAE (subcritical water extraction), obtaining high yields of FSPs at 127 °C, 15 min, and 25 mL of solvent per gram of macroalgae, together with 80 bar of pressure and 300 rpm of agitation speed. Moreover, hydrothermal technologies can also be used to hydrolyse macroalgal extracts aiming to estimate the amount of fucose when analysing FSPs. Thereby, Ozawa et al. [25] used an autoclave technology at 110 °C for 1 h to perform the acidic hydrolysis of extracts from *L. japonica*, while Sinurat et al. [26] hydrolysed polysaccharides from brown macroalgae (*Sargassum*, *Tubinaria,* and *Padina* species) using 3 N trifluoroacetic acid in an autoclave at 121 °C for 1 h.

The obtention of maximum yields of total glucans required milder extraction conditions (99.3 °C, 30 min, and 21.3 mL of solvent; condition 2, Table 5) than those previously described for fucose. Similarly to these results, Rajauria, Jaiswal, Abu-Ghannam and Gupta [19] obtained higher yields of total soluble sugars from brown macroalgae (*L. digitata*, *Laminaria saccharina,* and *Himanthalia elongata*) using HAE at 85 °C during 15 min when compared to other extracts generated at 100, 110, and 121 °C. However, other reports aiming to produce extracts rich in soluble sugars from fungi *Grifola frondosa* obtained high yields of total sugars using a HAE at 121 °C [27]. Moreover, previous reports aiming to achieve high yields of glucans from fungi and cereals reported variable yields and HAE conditions [28,29,30,31].

The optimised conditions for obtaining extracts with maximum antioxidant (FRAP and DPPH) activities were 120 °C, 76.06 min, and 10 mL of solvent per gram of macroalgae (condition 3; Table 5). Similarly to our results, Rajauria, Jaiswal, Abu-Ghannam and Gupta [19] determined that HAE at temperatures higher than 85 °C, needed to obtain high yields of total sugars, resulted in extracts with high antioxidant activity. The authors obtained extracts with high FRAP activities using HAE at 110 °C, while the extracts with the highest DPPH radical scavenging activities were generated at 95 °C [19].

The optimum conditions to achieve high yields of fucose, total glucans, and antioxidant activities were of temperature (120 °C), time (80.9 min) and volume of solvent (12.02 mL per g of macroalgae) as described in condition 4 (Table 5). To our knowledge there are no studies aiming to optimise the yields of fucose, total glucans, and antioxidant activities from macroalgae using HAE.

### 2.4. Exploring Optimum HAE to Recover Polysaccharides from Other Brown Macroalgae

The optimum extraction conditions (120 °C, 80.9 min, and 12.02 mL per gram of macroalgae; condition 4, Table 5) achieved high yields of fucose (2782.3 ± 70.1 mg/100 g ds) and total glucans (2344.1 ± 12.0 mg/100 g ds) from *L. hyperborea*. These extraction conditions, aiming to achieve high yields of both polysaccharides, were also explored in other brown macroalgae (*L. digitata* and *A. nodosum*). The content of fucose and total glucans of the extracts generated using this optimised extraction protocol were extremely variable depending on the macroalgal species. Extracts from *L. digitata* contained lower levels of fucose (943.0 ± 8.6 mg/100 g ds) and total glucans (219.5 ± 29.2 mg/100 g ds) than those from *L. hyperborea* (experimental values, condition 4, Table 4). In the case of *A. nodosum*, the extracts had higher fucose levels (6978.4 ± 20.2 mg/100 g ds) than those of *L. hyperborea* and *L. digitata,* and intermediate levels of total glucans (640.0 ± 13.6 mg/100 g ds) between those of the other two species of *Laminaria*. Similarly to these results, Rioux et al. [32] reported substantial differences in the concentration and chemical structure of polysaccharides extracted from *A. nodosum*, *Fucus vesiculosus,* and *Saccharina longicruris* using the same extraction protocol. Previous studies reported differences in the contents of polysaccharides and other macro-nutrients in macroalgae depending on a wide variety of factors such as the species, season, and location of collection [6,16,33]. All the macroalgal biomass from this study was collected in the same season and location, and the extraction performed using the same protocol; thus, the variation in the concentration of polysaccharides obtained in these extracts could be attributed to inter-species variability.

### 2.5. Sequential Application of Ultrasound and Thermal Technologies

One of the current trends in algal biotechnology involves the application of multiple processes or combinations of technologies, aiming to transform the algal biomass into a wide variety of high-value products while generating minimum waste, following a biorefinery concept [21]. Understanding the impact of all the process parameters on the efficiency of the treatment of the biomass and on the final yields of carbohydrates and other high-value compounds is a key critical point that will influence the design and future utilisation of macroalgae, following a biorefinery concept [22]. Thus, the macroalgal residues filtered from *L. hyperborea*, *L. digitata,* and *A. nodosum* after HAE were further processed to explore the effect of the sequential application of ultrasound and thermal technologies, using multiple time combinations (0, 15, and 30 min) to increase the recovery of fucose and total glucans.

**Figure 3:**
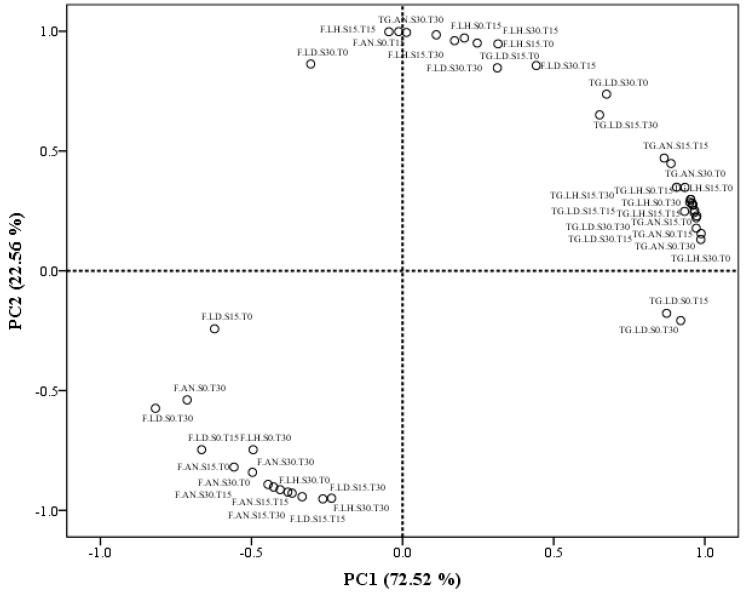
PCA scatter plot representing the scores for the recovery of fucose and total glucans from each macroalgae species and treatment used. Abbreviations in the figure are as follows: F (fucose), TG (total glucans), LH (*Laminaria hyperborea*), LD (*Laminaria digitata*), and AN (*Ascophyllum nodosum*). The technological treatments applied during 0, 15, and 30 min were abbreviated as follows: Sonication (S0, S15, and S30) and thermal treatment (T0, T15, and T30).

Principal component analysis (PCA) was performed to obtain an overview of the similarities and differences in the recovery of fucose and total glucans from the macroalgal residues of *L. hyperborea*, *L. digitata,* and *A. nodosum,* depending on the different combination of technologies applied. The two principal components, PC1 and PC2, obtained from the data explained 72.52 and 22.56% of the total variance in the data set, respectively (see Figure 3). PC1 is highly correlated with the recovery of total glucans, and most of these values clustered on the right side of PC1, indicating a close relationship between them. On the other hand, most of the recoveries of fucose were situated in close proximity to each other on the opposite side of PC1. The opposite behaviour of the recoveries of fucose and total glucans could indicate the need to implement different extraction approaches to recover both molecules separately from macroalgae when designing future biorefinery approaches from brown macroalgae. However, previous studies have also mentioned the difficulties when extracting glucans from brown macroalgae. Rioux et al. [32] reported an unavoidable co-extraction of FSPs together with glucans, remarking the need to perform a later fractionation of both polysaccharides at the expense of substantially reducing the yields of total glucans obtained [32].

The second component explained further the variability of the data set and seemed to be mainly associated with the recoveries of fucose. PC2 is positively correlated with few isolated recoveries of fucose, and the remaining total glucan recoveries that were not fully explained by the main cluster in PC1. This second component is negatively associated with the main cluster of fucose recoveries (see Figure 3). These variable results in the recovery of fucose could be explained by differences in the chemical structure of these molecules, depending on their function within the macroalgal cells. Thereby, FSPs located in the outer layer of the cell wall are mainly related to ion exchange properties, explaining the adaptation of macroalgae to salinity variations; while other FSPs chemical structures are mainly found in the cell walls, binding strongly with other compounds to configure the cell wall skeleton [34]. Moreover, previous studies have also reported a huge variation in the chemical structure (i.e., molecular weight, monosaccharide composition, and degree of sulphation) of FSPs extracted from multiple brown macroalgae species [16,32].

The recoveries of fucose and total glucans from the macroalgal residues of *L. hyperborea*, *L. digitata,* and *A. nodosum* using different ultrasound and thermal technologies sequentially during 0, 15, and 30 min are further explored in Table 5. The fucose and total glucans recovered were extremely variable depending on the macroalgal species. In general, the additional recoveries of fucose from both of the species of *Laminaria* were low (*L. hyperborea*: 487.4 ± 10.3 mg fucose/100 g dried macroalgal residue (dmr); *L. digitata*: 155.1 ± 1.0 mg/100 g dmr) compared to *A. nodosum* (2971.7 ± 61.9 mg fucose/100 g dmr). The thermal treatment seems to play little or no effect on the extraction yields of fucose from the two species of *Laminaria*, as the maximum recoveries were obtained by applying 30 min of sonication water bath and no thermal treatment. However, in the case of *A. nodosum,* the maximum recovery of fucose was obtained by applying sequential sonication and thermal treatments during 30 min. These variable results on the recovery of fucose amongst both *Laminaria* species and *A. nodosum* could be attributed to the differences in structural and chemical features of FSPs produced by these species [34]. Previous studies have reported that macroalgae from the genus *Laminaria* contain mainly sulphated homofucans, located in the intercellular matrix of the cell walls; while algae from the genus *Ascophyllum* predominantly produce heterogeneous sulphated fucans or ascophyllans that could play a crucial role in the cell wall by binding strongly to alginates and cellulose configuring a cell wall skeleton [35].

The maximum recoveries of total glucans were obtained from *L. hyperborea* (908.0 ± 51.4 mg/100 g dmr), followed by *A. nodosum* (494.2 ± 26.9 mg/100 g dmr) and *L. digitata* (134.8 ± 11.8 mg/100 g dmr). As seen in Table 6, the conditions to recover total glucans were extremely variable depending on the macroalgal species. The recovery of total glucans from *L. hyperborea* and *L. digitata* did not show a clear combination of technologies to achieve outstanding recoveries. For example, in *L. hyperborea*, the application of sonication (15 min) followed by thermal treatment (30 min) obtained similar recoveries to the application of sonication for 30 min. However, the application of either a thermal or a sonication treatment for 30 min achieved high recoveries of total glucans from *L. digitata*. In the case of *A. nodosum*, the maximum recoveries of total glucans were achieved by using sonication and thermal technologies sequentially during 30 min, similar to the case of fucose. This variability in the recovery of total glucans could be attributed to the chemical structure of the glucans produced by different macroalgal species. β-glucans or laminarin are the main glucan or storage carbohydrate of brown macroalgae [6,36]. The chemical structure of β-glucans can be described as a main chain of 1,3-linked β-d-glucose residues with different degrees of branching at β-(1,6) which influence the water solubility properties of these polysaccharides [6]—i.e., variable contents of water-soluble and insoluble laminarin were obtained in *L. digitata* and *L. hyperborea* [10,37]. Moreover, the degree of branching and the presence or absence of terminal mannitol residues may also vary depending on the macroalgae species, but also on the physiological and environmental conditions affecting the biomass [10].

## 3. Materials and Methods

### 3.1. Macroalgae Biomass and Proximate Composition Analyses

*L. hyperborea*, *L. digitata,* and *A. nodosum* were harvested in February 2016 (Quality Sea Veg Ltd., Co. Donegal, Ireland). Samples were cleaned from epitopes, oven-dried following industry practices (50 °C, 9 days), and milled to 1 mm particle size using a hammer mill (Christy and Norris, Chelmsford, UK). All the samples were vacuum-packed and stored at room temperature for further analyses. The dry matter of the dried and milled macroalgae was determined by oven-drying the samples at 105 °C for 16 h. The ash content was determined after ignition of a weighed sample in a muffle furnace at 550 °C for 6 h according to the AOAC.942.05 [38]. The N content was determined using the LECO FP 528 instrument (Leco Instruments UKLTD., Cheshire, UK), using the conversion factor 4.17 as described for brown macroalgae by Biancarosa et al. [39]. The ether extract was determined using Soxtec instrumentation (Tecator, Sweden) following the AOAC.920.39 [38], and the total soluble sugars were estimated following the phenol-sulfuric acid assay as described by Brummer and Cui [40]. The measurements of fucose and total glucans were performed following the methodology described in Section 3.5.

### 3.2. Chemicals

Citric acid, sodium acetate, ferric chloride, sodium phosphate dibasic, methanol, triton™ X-100, sulfuric acid (95–97%), L-cysteine, potassium hydroxide, hydrochloric acid, and standards (6-hydroxy-2,5,7,8-tetramethylchromane-2-carboxylic acid (trolox), 1,1-diphenyl-2-picryl-hydrazyl (DPPH)), 2,4,6-tripyridyl-s-triazine (TPTZ) L-(-)-Fucose and ascorbic acid) were purchased from SIGMA (Sigma-Aldrich, St. Louis, MO, USA). Acetic acid and sodium hydroxide were purchased from VWR (VWR International, Radnor, PA, USA) and the enzymatic glucan assay kits K-YBGL were purchased from Megaenzyme International Ltd., Bray, Ireland. All other reagents used were of analytical grade.

### 3.3. Hydrothermal-Assisted Extraction (HAE)

The scheme of work followed to optimise HAE parameters using *L. hyperborea* is represented in Figure 1. The extraction was performed using 10 g macroalgae, mixed with different volumes of 0.1 M HCl (10–30 mL solvent per gram of macroalgae) and stirred for 10 min before starting the HAE. The selection of 0.1 M HCl was based on previous reports emphasising the use of this solvent to increase the yields of polysaccharides from various macroalgae [8,18].

The HAE was performed using an autoclave apparatus (Tomy SS-325; Tomy Seiko Co. Ltd., Tokyo, Japan). The extraction variables temperature (°C), time (min), and volume of solvent (mL/g macroalgae) were optimised for *L. hyperborea* using multiple HAE conditions as described in detail in Section 3.7. Each extraction condition was performed in duplicate. The macroalgal residues were filtrated through Whattman^®^ number 3 (Sigma-Aldrich) and the supernatants combined. The combined extracts were freeze-dried in an industrial scale freeze-drier (FD80 model 119, Cuddon Engineering, Blenheim, New Zealand), vacuum sealed and stored at −20 °C until further analysis.

The optimum HAE conditions designed for *L. hyperborea* were further tested in other brown macroalgae species (*L. digitata* and *A. nodosum*). The macroalgal extracts were processed following the same procedures as described for *L. hyperborea*. All the macroalgal residues were dried and preserved for further extraction.

### 3.4. Sequential Application of Ultrasound and Thermal Extraction Technologies

The macroalgal residues from *L. hyperborea*, *L. digitata,* and *A. nodosum* were re-dissolved in 0.1 M HCl (10 mL solvent per g macroalgae) and extracted again using ultrasound and thermal technologies sequentially.

The ultrasound extraction was performed using a sonication water bath at 80 W of power and 50–60 Hz of ultrasonic frequency (Fisherbrand™, Fisher Scientific, Schwerte, Germany). The thermal extraction was performed in a shaking water bath at 200 rpm and 100 °C (WBS-30, Daihan Scientific Co., Ltd., Seoul, Korea). Both extraction technologies were applied using different time combinations of 0, 15, and 30 min. Each extraction combination was performed in duplicate, filtered, and the extracts were combined, freeze-dried, and stored at −20 °C to analyse the recovery of fucose and total glucans obtained with each combination of technologies.

### 3.5. Polysaccharide Measurements

The FSPs of the samples are estimated by measuring the concentration of fucose in the algal extracts, and the concentration of total glucans was measured using an enzymatic protocol. All the measurements were performed in duplicate.

#### 3.5.1. Fucose Estimation

The concentration of fucose as an estimation of the FSPs or fucoidans was performed according to the method described by Usov et al. [41], with the modifications previously described by Garcia-Vaquero et al. [8], Skriptsova [42], and Voron’ko et al. [43]. Briefly, the fucose standards (0.005 to 0.1 mg/mL) and extracts (diluted to fit the calibration points) were hydrolysed using concentrated sulfuric acid in water (6:1, H_2_SO_4_:H_2_O) for 10 min at 100 °C in a water bath. The samples were derivatised using cysteine hydrochloride and the absorbance of the reaction read at 396 and 430 nm using a microplate reader (Epoch, BioTek, Winooski, VT, USA). The fucose values are expressed as mg fucose per 100 g of dried macroalgae or residue.

#### 3.5.2. Total Glucans

The total glucans were determined using the kit K-YBGL, purchased from Megaenzyme (Bray, Ireland), following the manufacturer’s recommendations. Briefly, 100 mg of sample and positive control (yeast β-glucan) were digested in concentrated HCl (37% *w*/*v*) at 30 °C for 45 min, diluted, and hydrolysed using a water bath (100 °C, 100 rpm, and 2 h). Samples were neutralised with 2 M KOH and adjusted to 100 mL with sodium acetate buffer (pH 5.0), and centrifuged (1000 g, 10 min). All the samples were then enzymatically treated using a solution containing exo-1,3-β-glucanase (20 units (U)/mL) and β-glucosidase (4 U/mL), followed by the addition of glucose-oxidase-peroxidase-reagent (GOPOD). The absorbance of the samples, blanks, and glucose standards was read at 510 nm in a spectrophotometer (UVmini-1240, Shimadzu, Japan). The total glucan concentrations are expressed as mg total glucans per 100 g of dried macroalgae or residue.

### 3.6. Antioxidant Activity Measurements

The antioxidant capacity of the macroalgal extracts was performed by measuring the ferric reducing antioxidant power (FRAP) and the 1,1-diphenyl-2-picryl-hydrazyl (DPPH) radical scavenging activity of the samples. All the measurements were performed in duplicate.

#### 3.6.1. Ferric Reducing Antioxidant Power (FRAP)

The FRAP was assayed using the method described by Benzie and Strain [44], modified by Bolanos de la Torre et al. [45]. The macroalgal extracts were diluted at 1 mg/mL and assayed against trolox standards (15–420 µM) using a working solution containing acetate buffer (300 mM, pH 3.6), ferric chloride (20 mM in Milli Q water), 2,4,6-Tripyridyl-s-Triazine (TPTZ) (10 mM in 40 mM HCl), and Milli Q water. The reaction was performed in a Greiner CELLSTAR^®^ 96 incubated at 37 °C for 30 min, and the absorbance of the reaction was read at 593 nm. FRAP values are expressed as µM trolox equivalents per mg of freeze-dried extract.

#### 3.6.2. 1,1-Diphenyl-2-Picryl-Hydrazyl (DPPH) Radical Scavenging Activity

The DPPH assay was performed following the method proposed by Nicklisch and Waite [46], with the modifications previously described by Garcia-Vaquero, Rajauria, Tiwari, Sweeney and O’Doherty [8]. Briefly, samples and positive control (ascorbic acid) were diluted to 1 mg/mL in 0.1 M citrate phosphate buffer containing 0.3% of Triton X-100. The reaction was started by adding 10 µL of a 2 mM methanolic DPPH solution to each well, and the % of DPPH inhibitory activity was calculated by subtracting the absorbance readings of the wells at 515 nm before and after 30 min of starting the chemical reaction.

### 3.7. Experimental Design and Statistical Analyses

The optimisation of the extraction variables using HAE to obtain high yields of polysaccharides and antioxidant activities from *L. hyperborea* was performed by applying response surface methodology (RSM) using a Box-Behnken design (BBD) with three independent variables at four levels. The experimental order was randomised and the levels of the independent variables temperature (80–120 °C), time (30–90 min), and volume of solvent (10–30 mL/g macroalgae) were coded as described in Table 7.

The experimental design matrix and the extraction yields of fucose (mg/100 g dm), total glucans (mg/100 g dm), FRAP (µM trolox/mg fde), and DPPH (% radical scavenging activity) are summarised in Table 1 previously discussed in Section 2.1. The results were analysed using response surface regression (SAS version 9.2) and fitted to a second-order polynomial model represented in Equation (5).

(5) $$Y=\;\beta 0+\overset{3}{\underset{i=1}{\sum }}\beta_{i}X_{i}\;\overset{3}{\underset{i=1}{\sum }}\beta_{\mathrm{ii}}X_{i}^{2}+\;\overset{3-1}{\underset{i}{\sum }}\;\overset{3}{\underset{j}{\sum }}\beta_{\mathrm{ij}}X_{i}X_{j}$$

where, Y represents the predicted response for fucose, total glucans, FRAP and DPPH; X_i_ and X_j_ are the independent variables; and β represents coefficients: β_0_ (constant coefficient), β_i_ (linear coefficient), β_ii_ (quadratic coefficient), and β_ij_ (cross product coefficients).

The mixed plots containing contour (2D) and response surface (3D) were generated using Design Expert (v.11) software to represent the variation in the experimental responses attained with multiple combinations of two independent variables while holding one of the components constant in the second-order polynomial model. The validity of the optimised model was determined by comparing the experimental and predicted values (Table 5, Section 2.3).

After the optimisation process, the macroalgal residues from *L. hyperborea*, *L. digitata,* and *A. nodosum* were further extracted using ultrasound and thermal technologies to explore the recovery of fucose and total glucans. The main variance in the data was analysed by principal component analysis (PCA) in SPSS version 24. The PCA was performed using the Varimax rotation method with Kaiser normalisation to obtain the expected weight for each component extracted from the matrix with eigenvalues higher than 1. The recoveries of polysaccharides were further analysed using multivariate general lineal model. There was a significant (*p* < 0.001) influence of the macroalgae species, ultrasound, and thermal treatments on the recoveries of both fucose and total glucans. These differences were further analysed statistically by using Student’s *t*-tests or Tamhane post-hoc tests.

## 4. Conclusions

HAE was explored using response surface methodology to elucidate the effects of the extraction variables (temperature, time, and volume of solvent) on the yields of fucose, total glucans, and antioxidant activities (FRAP and DPPH) from the macroalgal species *L. hyperborea*. Optimised conditions of 120 °C, 80.9 min, and 12.02 mL of 0.1 M HCl per gram of macroalgae achieved 2782.3 mg fucose/100 g dm, 2344.1 mg total glucans/100 g dm, 54.7 ± 0.4 µM trolox/mg fde, and 45.4% of DPPH radical scavenging activity. The HAE conditions to recover polysaccharides and antioxidant activities from *L. hyperborea* were applied to other brown macroalgae (*L. digitata* and *A. nodosum*), and the algal residues were further processed by sequential extraction with ultrasound and thermal technologies using multiple time combinations (0, 15, and 30 min) to explore the recoveries of polysaccharides. The recovery of fucose and total glucans was extremely variable depending on the macroalgal species. The maximum recoveries of fucose were obtained from *A. nodosum* (2971.7 ± 61.9 mg fucose/100 g dmr) using 30 min of sonication and 30 min of thermal treatment; while the maximum recovery of total glucans was extracted from *L. hyperborea* (908.0 ± 51.4 mg total glucans/100 g dmr) applying an ultrasound treatment (15 min) followed by thermal extraction (30 min). Moreover, data from principal component analysis revealed an opposite behaviour of the recoveries of fucose and total glucans when using multiple technologies, indicating the need to implement different extraction approaches to recover both molecules separately from macroalgae when designing future biorefinery approaches from brown macroalgae. Future studies will be needed in order to purify and characterise fully the FSPs and glucans fractions obtained using these optimised conditions.

## Figures and Tables

**Table 1:** Proximate composition (dry matter, ash, protein, crude lipids, total glucans, and fucose) of the dried macroalgae used in this study.

Proximate Composition	Macroalgae Species		
	*L. hyperborea*	*L. digitata*	*A. nodosum*
Dry matter (%)	90.83 ± 0.00	91.39 ± 0.01	90.38 ± 0.01
Ash (%)	30.01 ± 0.03	33.84 ± 0.08	23.31 ± 0.3
Protein (% DW basis)	9.98 ± 0.01	11.12 ± 0.76	6.14 ± 0.01
Ether extract (% DW basis)	0.76 ± 0.07	0.26 ± 0.05	3.33 ± 0.00
Total soluble sugars (% DW basis)	14.49 ± 0.11	11.88 ± 0.13	13.66 ± 0.08
Total glucans (% DW basis)	6.40 ± 0.09	1.51 ± 0.02	2.70 ± 0.08
Fucose (% DW basis)	2.66 ± 0.03	0.77 ± 0.09	10.09 ± 0.09

The results are expressed as mean ± standard deviation of the mean (*n* = 3). The units are expressed as % DW (dry weight) basis.

**Table 2:** Experimental design and responses obtained to optimise hydrothermal-assisted extraction.

Run Order	Extraction Variables			Experimental Responses ^γ^			
	Temperature (°C)	Time (min)	Volume Solvent (mL/g Macroalgae)	Fucose	Total Glucans	FRAP	DPPH
1	120	60	10	2115.43 ± 58.12	2621.50 ± 9.97	59.64 ± 2.63	45.34 ± 0.89
2	120	60	30	3216.39 ± 31.95	2800.24 ± 348.88	29.40 ± 1.86	14.69 ± 0.44
3	100	30	30	2023.87 ± 124.93	2951.70 ± 24.33	17.34 ± 0.82	20.44 ± 1.26
4	100	90	10	1776.03 ± 90.44	2237.41 ± 103.60	24.61 ± 0.57	24.98 ± 1.37
5	80	90	20	1957.96 ± 135.15	2845.70 ± 99.51	22.80 ± 0.58	34.63 ± 1.26
6	80	30	20	1629.18 ± 123.53	2863.38 ± 64.11	22.85 ± 0.87	23.95 ± 3.23
7	100	60	20	2118.09 ± 84.24	2939.18 ± 3.99	22.14 ± 1.27	15.67 ± 1.83
8	80	60	30	1904.81 ± 23.99	2709.51 ± 313.70	19.68 ± 1.51	30.43 ± 1.27
9	100	60	20	2539.09 ± 128.55	3139.47 ± 31.78	24.78 ± 0.69	11.69 ± 1.33
10	80	60	10	1161.06 ± 51.29	1571.88 ± 154.36	23.46 ± 1.12	36.79 ± 1.21
11	120	30	20	2427.49 ± 163.58	2826.53 ± 146.26	23.38 ± 0.34	16.25 ± 0.64
12	120	90	20	2663.70 ± 142.20	3142.40 ± 42.11	35.27 ± 0.62	36.85 ± 1.29
13	100	30	10	1584.90 ± 33.39	2419.58 ± 45.50	22.92 ± 2.14	33.54 ± 1.38
14	100	90	30	1998.96 ± 135.04	2490.35 ± 26.61	20.37 ± 0.28	20.51 ± 1.29
15	100	60	20	2480.08 ± 222.08	3325.41 ± 15.77	22.85 ± 0.45	18.29 ± 4.13
16	100	60	20	2423.92 ± 109.84	3181.43 ± 8.69	24.99 ± 0.47	21.07 ± 1.77
17	100	60	20	2276.02 ± 87.19	2869.92 ± 11.17	24.71 ± 0.40	17.93 ± 2.00

^γ^ All the experimental responses are expressed as mean ± standard deviation of the mean (*n* = 6). The units of the experimental responses are expressed as follows: Fucose (mg/100 g dried macroalgae), total glucans (mg/100 g dried macroalgae), FRAP (µM trolox/mg freeze-dried extract), and DPPH (% radical scavenging activity).

**Table 3:** Analysis of variance showing the effect of the hydrothermal-assisted extraction variables on the response variables (fucose, total glucans, FRAP, and DPPH).

	Response Variables			
Coefficient	Fucose	Total Glucans	FRAP	DPPH
Linear	10.25 **	2.04	7.45 *	3.63 ^a^
Quadratic	2.31	11.06 **	3.25 ^a^	4.32 ^a^
Cross product	0.15	2.35	2.22	1.51
Lack of fit (*p*)	0.0269	0.0781	0.0018	0.0491
Total model	4.24 *	5.15 *	4.31 *	3.15 ^a^
RSME	281.29	295.1	5.63	6.48
CV	14.65	11.37	21.68	26.05
R^2^	0.8449	0.8688	0.8471	0.8022

^a^ Tendency towards significance at *p* < 0.1. * Statistically significant at *p* < 0.05. ** Statistically significant at *p* < 0.01.

**Table 4:** Regression coefficients and ANOVA of the predicted response surface quadratic models.

	Response Variables ^γ^			
Coefficient ^Ϸ^	Fucose	Total Glucans	FRAP	DPPH
β_0_	−54.25	−12,682 *	122.57	261.47 *
	(4001.5)	(4198)	(−80.05)	(92.23)
**Linear**				
β_1_	−25.45	233.37 *	−2.75 ^a^	−4.20 *
	(71.54)	(75.05)	(1.43)	(1.65)
β_2_	47.72	−40.74	0.19	−0.73
	(31.34)	(32.88)	(0.63)	(0.72)
β_3_	48.03	471 **	1.81	−0.44
	(94.03)	(98.65)	(1.88)	(2.17)
**Quadratic**				
β_11_	0.19	−1.03 *	0.017 *	0.022 *
	(0.34)	(0.36)	(0.007)	(0.008)
β_22_	−0.35 ^a^	0.086	−0.0053	0.0022
	(0.15)	(0.16)	(0.003)	(0.0035)
β_33_	−1.43	−6.95 **	0.022	0.06
	(1.37)	(1.44)	(0.03)	(0.03)
**Cross product**				
β_12_	−0.0093	0.30	0.005	0.004
	(0.23)	(0.25)	(0.0047)	(0.0054)
β_23_	−0.09	−0.076	0.001	0.007
	(0.47)	(0.49)	(0.009)	(0.01)
β_13_	0.45	−1.73 ^a^	−0.03 ^a^	−0.03
	(0.70)	(0.74)	(0.014)	(0.016)

^γ^ The units of the experimental responses are expressed as follows: Fucose (mg/100 g dried macroalgae), total glucans (mg/100 g dried macroalgae), FRAP (µM trolox/mg freeze-dried extract), and DPPH (% radical scavenging activity). ^Ϸ^ Estimated coefficients of the model are: β_0_ (constant coefficient), linear regression coefficients (β_1_, β_2_ and β_3_), and quadratic (β_11_, β_22_ and β_33_) and interaction (β_12_, β_23_, β_13_) effects of the model referred to the variables X_1_ (temperature), X_2_ (time), and X_3_ (volume of solvent). ^a^ Tendency towards significance at *p* < 0.1. * Statistically significant at *p* < 0.05. ** Statistically significant at *p* < 0.01.

**Table 5:** Optimum hydrothermal-assisted extraction conditions, predicted values, and experimental responses obtained of fucose, total glucans, and antioxidant activities (FRAP and DPPH).

Optimum Conditions	Targeted Bioactive Compounds ^γ^	Parameters of Extraction			Predicted Values (95% CI) ^a^	Experimental Response (mean ± SEM) ^b^
		Temperature (°C)	Time (min)	Volume Solvent (mL/g Macroalgae)		
Condition 1	Fucose	120	62.1	30	Fucose (2308, 3462)	Fucose (3132.3 ± 100.1)
Condition 2	Total glucans	99.3	30	21.3	Total glucans (2800, 3643)	Total glucans (2825.7 ± 5.6)
Condition 3	FRAP	120	76.06	10	FRAP (41.20, 66.12)	FRAP (44.3 ± 0.4)
	DPPH				DPPH (31.05, 59.76)	DPPH (42.6 ± 1.6)
Condition 4	Fucose	120	80.9	12.02	Fucose (1562, 2716)	Fucose (2782.3 ± 70.1)
	Total glucans				Total glucans (2117, 3328)	Total glucans (2344.1 ± 12.0)
	FRAP				FRAP (38.74, 61.83)	FRAP (54.7 ± 0.4)
	DPPH				DPPH (28.57, 55.17)	DPPH (45.4 ± 0.6)

^a^ The predicted values were expressed as 95% confidence intervals. ^b^ Experimental responses were expressed as mean ± standard deviation of the mean. Number of readings (*n* = 6). ^γ^ The units of the experimental responses are expressed as follows: Fucose (mg/100 g dried macroalgae), total glucans (mg/100 g dried macroalgae), FRAP (µM trolox/mg freeze-dried extract), and DPPH (% radical scavenging activity).

**Table 6:** Additional recoveries of fucose and total glucans from the residual biomass obtained after hydrothermal-assisted extraction. The application of sequential ultrasound and thermal technologies was explored using multiple time combinations (0, 15, and 30 min).

Compounds Recovered ^γ^	Macroalgae sp.	Ultrasound Treatment (min)	Thermal Treatment (min)		
			0	15	30
Fucose	*L. hyperborea*	0	-	299.3 ± 8.5 (B, b)	330.8 ± 1.5 (A, c)
		15	238.9 ± 9.5 (C, b)	314.9 ± 0.8 (B, b)	460.6 ± 8.8 (A, a)
		30	487.4 ± 10.3 (A, a)	344.2 ± 4.4 (B, a)	307.3 ±1.8 (C, b)
	*L. digitata*	0	-	146.79 ± 2.0 (A, a)	150.8 ± 1.4 (A, a)
		15	120.4 ± 0.6 (B, b)	125.38 ± 2.1 (B, b)	143.7 ± 0.6 (A, a)
		30	155.1 ± 1.0 (A, a)	151.05 ± 2.1 (A, a)	128.2 ± 1.6 (B, b)
	*A. nodosum*	0	-	1569.0 ± 7.8 (B, c)	1655.5 ± 6.6 (A, b)
		15	1634.1 ± 27.8 (B, a)	1995.1 ± 51.7 (A, a)	1741.3 ± 36.2 (AB, b)
		30	1411.0 ± 25.4 (B, b)	1312.7 ± 28.0 (B, b)	2971.7 ± 61.9 (A, a)
Total glucans	*L. hyperborea*	0	-	594.2 ± 37.6 (A, a)	543.9 ± 34.8 (A, b)
		15	314.6 ± 21.2 (C, b)	629.5 ± 45.4 (B, a)	908.0 ± 51.4 (A, a)
		30	900.7 ± 51.4 (A, a)	700.7 ± 43.5 (AB, a)	657.5 ± 45.0 (B, b)
	*L. digitata*	0	-	93.0 ± 6.3 (B, a)	134.8 ± 11.8 (A, a)
		15	99.8 ± 14.4 (A, a)	87.2 ± 13.1 (A, a)	93.7 ± 7.3 (A, a)
		30	127.6 ± 9.0 (A, a)	103.3 ± 12.2 (A, a)	103.7 ± 15.3 (A, a)
	*A. nodosum*	0	-	269.04 ± 17.9 (A, ab)	255.5 ± 20.7 (A, b)
		15	253.0 ± 21.9 (A, a)	319.55 ± 18.7 (A, a)	288.9 ± 19.2 (A, b)
		30	220.9 ± 13.7 (B, a)	202.6 ± 15.6 (B, b)	494.2 ± 26.9 (A, a)

Different uppercase letters represent the statistical differences (*p* < 0.05) in the recoveries of fucose and total glucans between thermal treatment times (0, 15, and 30 min) while receiving the same ultrasound treatment. The different lowercase letters indicate the statistical differences (*p* < 0.05) in the recoveries of fucose and total glucans between ultrasound treatments (0, 15, and 30 min) while receiving the same thermal treatment. ^γ^ The recovery of compounds is expressed as fucose (mg/100 g dried macroalgal residue) and total glucans (mg/100 g dried macroalgal residue).

**Table 7:** Independent variables and codes used for the optimisation of HAE.

Independent Variables	Symbols	Coded Levels		
		−1	0	+1
Temperature (°C)	X1	80	100	120
Time (min)	X2	30	60	90
Solvent (mL/g macroalgae)	X3	10	20	30
